# Chinese Herbal Therapy and Western Drug Use, Belief and Adherence for Hypertension Management in the Rural Areas of Heilongjiang Province, China

**DOI:** 10.1371/journal.pone.0123508

**Published:** 2015-04-29

**Authors:** Xia Li, Mingkai Peng, Yuan Li, Zheng Kang, Yanhua Hao, Hong Sun, Lijun Gao, Mingli Jiao, Qunhong Wu, Hude Quan

**Affiliations:** 1 Department of Social Medicine, School of Public Health, Harbin Medical University, Harbin, China; 2 Department of Community Health Sciences, University of Calgary, Calgary, Alberta, Canada; 3 College of Pharmacy, Harbin Medical University, Harbin, China; The Ohio State University, UNITED STATES

## Abstract

**Background:**

Traditional Chinese medicine (TCM) including Chinese herbal therapy has been widely practiced in China. However, little is known about Chinese herbal therapy use for hypertension management, which is one of the most prevalent chronic conditions in China. Thus we described Chinese herbal therapy and western drug users, beliefs, hypertension knowledge, and Chinese herbal and western drug adherence and determinants of Chinese herbal therapy use among patients with hypertension in rural areas of Heilongjiang Province, China.

**Methodology and Principal Findings:**

This face-to-face cross sectional survey included 665 hypertensive respondents aged 30 years or older in rural areas of Heilongjiang Province, China. Of 665 respondents, 39.7% were male, 27.4% were aged 65 years or older. At the survey, 14.0% reported using Chinese herbal therapy and 71.3% reported using western drug for hypertension management. A majority of patients had low level of treatment adherence (80.6% for the Chinese herbal therapy users and 81.2% for the western drug users). When respondents felt that their blood pressure was under control, 72.0% of the Chinese herbal therapy users and 69.2% of the western drug users sometimes stopped taking their medicine. Hypertensive patients with high education level or better quality of life are more likely use Chinese herbal therapy.

**Conclusions and Significance:**

Majority of patients diagnosed with hypertension use western drugs to control blood pressure. Chinese herbal therapy use was associated with education level and quality of life.

## Introduction

Traditional Chinese medicine (TCM) has a long history and includes Chinese herbal therapy, massage, scrapping, cupping, and acupuncture. In China, integration of TCM and western medicine is a national healthcare policy[1]. The Chinese government sponsors TCM universities or colleges for training TCM physicians, TCM hospitals for providing special services and TCM departments in western medicine hospitals for integration.

Chinese herbal therapy is widely utilized in China and reputed and perceived as being safe, being cost-effective and having no side-effect. Quan et al. [2] reported that 66% of Chinese Canadians even believe that herbal therapy could cure a chronic disease and 70% believe that herbal therapy has been scientifically tested to be effective. A survey conducted by Fang [3] in China shows that 38.3% of the Chinese respondents used TCM for chronic disease management. Zhao et al. [4] reported that the characteristics of TCM users in China are predominantly urban residents, females, and have high educational level, and high income.

Hypertension is one of the most prevalent chronic conditions in China (18.8% prevalence rate), affecting about 200 million patients [5–6]. Of those, 110 million patients reside in rural areas. Hypertension-related diseases cost 31.89 billion Yuan (about 4.8 billion US dollars) per year, which accounts for 5.6% of the total health expenditure [7–8]. The essence of Chinese herbal therapy is syndrome differentiation, diagnoses syndromes with clinical manifestations and treats patients using the holistic approach by adjusting the various aspects of the body to restore the order and harmony of the body. In TCM, hypertension is classified by syndromes, including Gan-fire flaming-up syndrome, Yin-deficiency and Yang-excess syndrome, phlegm-dampness accumulation syndrome, and yin-yang deficiency syndrome [9–10]. Based on TCM principles various Chinese herbal therapies have been invented [10–11], and clinical guidelines have been developed[11–12]. For example Tian-Ma-Gou-Teng-Yin (Chinese herbal formula) is commonly used as an antihypertensive therapy. Effect of the therapy onsets in 30 minutes, reaches the highest in 60 minutes and lasts up-to 4 hours[13]. At present, the Chinese herbal formula is manufactured as tablets and available in markets as non-prescription medicine. However, little is known about TCM use for blood pressure control. Thus we conducted a face to face interview in rural areas of Heilongjiang Province, China. This study was aimed to describe Chinese herbal therapy and western drug users, beliefs, hypertension knowledge, and adherence of Chinese herbal therapy and western drug and to determine factors associated with Chinese herbal therapy use among patients with hypertension in rural areas of Heilongjiang Province, China.

## Materials and Methods

### Ethics Statement

All the participants in this study obtained and signed the informed consents. And this study was approved by the Harbin Medical University Ethics board.

### Study Population

This cross-sectional face to face interview survey included patients diagnosed with hypertension in two counties (i.e. Fujin and Linkou) of Heilongjiang province, China. Towns in each county were categorized into two groups based on economic development status (i.e. relatively poor and non-poor). Two convenient towns were selected from each economic status. In each town, villages with populations of at least 800 were classified into low, middle and high economic development groups and one convenient village was chosen from each group. Economic development level was determined by county health department staff who coordinated this survey and were familiar with economic status in the county.

Patients with hypertension and age ≥30 years were identified through existing and on-going hypertension registries in the 24 villages of the two counties. Generally, there is one physician resides in each village and is responsible for regular physical check-up of residents in the village. Physicians in the 24 villages were paid by fee-for-service and incented by number of hypertensive patients who were registered and managed. Thus the registry is relatively complete and frequently updated. We discussed our study population criteria with village physicians who registered hypertensive patients. These physicians reside in the villages and know residents well. Nearly all residents are farmers. Physicians are required to use to Joint National Committee on High Blood Pressure -7 (JNC-7) criteria [14] to define hypertension (systolic blood pressure of 140 mmHg or greater, and/or diastolic blood pressure of 90 mm Hg or greater, and/or self-reported current treatment for hypertension with antihypertensive medication). We excluded patients with secondary hypertension (such as pregnancy induced hypertension), stroke, senile dementia, severe mental disorder, or language barriers.

### Data Collection

The survey was conducted through face to face interview by undergraduate medical students during July 23 to 26, 2010 at the village clinics and administration offices. All interviewers received training and practiced the interview prior to the actual face-to-face interviews. The questions asked during the interview was tested among 25 people and revised according to feedback provided (the questionnaire is available on request).

Village physicians invited patients with hypertension to participate in the survey. Before interviewing, interviewers explained the purpose, confidentiality of the survey and subsequently invited patients to participate. Their participation in the interview was voluntary and accepted as oral consent. If there were more than one eligible person within a family, the individual to first arrive to the interview site was interviewed. During the interview, interviewers measured blood pressure and collected data using a questionnaire guide. The completeness of the interviews was checked right after the survey. If there was missing information, individuals were resurveyed.

### Study Variables

Chinese herbal therapy and western drug use was determined through asking participants a close-ended multiple choice question: "How do you control your blood pressure now?" Possible answers included western drug, Chinese herbal therapy, diet, acupuncture, physical activity, others (required to specify) and none. Information on dosage and frequency for each western drug or Chinese herbal therapy in the past two weeks was collected. None of the respondents used both herbal therapy and western drug at the time of interview in our sample. Some patients used Chinese herbal therapy and other treatment modalities (such as diet, acupunctures) at the same time. Those patients were classified as Chinese herbal therapy users. Non-users did not use Chinese herbal therapy and western drug but might use other treatment modality such as acupuncture.

We collected information on socio-demographic characteristics, self-reported physical and mental health status; physician diagnosed chronic diseases, and EuroQol-5 (EQ5). The EQ-5 has been validated in different countries and languages [15–17]. Physician diagnosed chronic diseases included heart disease, liver disease, peptic ulcer disease, renal disease, arthritis, chronic back-pain, diabetes, neurological disorder including stroke, cancer, allergy, and depression. Chronic disease referred to the above conditions diagnosed by physicians in the last 6 months before the interview, or diagnosed more than 6 months before the interview but reoccurred within the last 6 months and the patient had received treatment. Non physician-diagnosed chronic diseases were not included because the validity of self-diagnosed medical conditions depends on the level of the respondent’s knowledge and their perceptions on the definition of “disease” and “health”. Hypertension knowledge was assessed using an instrument developed by William [18] and the instrument has been used in previous studies[19–20]. The instrument contains 10 questions. Level of hypertension knowledge was defined at the 100-point-scale with a score of 10 for a correct answer for a question. Beliefs about Chinese herbal therapy were assessed using four-level Likert scale based on common perceptions on Chinese herbal therapy (Agree, Some Agree, Disagree, and Undecided). Treatment adherence of Chinese herbal therapy and western medicine was assessed using the ^***©***^MMAS-8 (Morisky Medication Adherence Scale, 8-Items) at a scale of 8 with 1 score for a correct answer to a question. [21]The English instrument was translated into Chinese following the sequential translation method by four persons who are bilingual [22].

### Statistical Analysis

“Descriptive statistics were employed to characterize baseline characteristics of patients with hypertension. The extent of Chinese herbal therapy and western drug use was stratified by socio-demographic characteristics, presence of chronic disease and quality of life. We employed F-test for continuous variables and chi-square test for categorical variables to examine statistical significance for differences among three groups (i.e. Chinese herbal therapy users, western drug users and non-users). Logistic regression was used to determine factors associated with Chinese herbal therapy use (dependent variable). Independent variables included age group, sex, employment, education, marital status, 11 chronic diseases, EQ5, herbal therapy belief, 10 hypertension knowledge questions, and Chines herbal therapy and western drug adherence level. All analyses were performed with SAS 9.1 statistical software (SAS Institute, Cary, NC).

## Results

Of the 665 respondents with diagnosed hypertension, 39.7% were males, 27.4% were aged 65 years or older, 85.7% were married, 23.9% were illiterates, 57.3% had chronic diseases and 58.9% had pain or discomfort problems (Table 1).

**Table 1:** Characteristics of patients with diagnosed hypertension.

Variable	n (% of 665)
Male	264(39.7)
Age (years)	
30–49	96 (14.4)
50–64	387(58.2)
≥65	182(27.4)
Married	570(85.7)
Education	
Illiteracy	159(23.9)
Elementary school	355(53.4)
Junior high school or higher	151(22.7)
Presence of any one of 11 chronic diseases*	381 (57.3)
Quality of life (EuroQol-5)	
Have walking problems	165 (24.8)
Have washing and dressing problems	72 (10.8)
Have problems with usual activities	153 (23.0)
Have pain or discomfort	392 (58.9)
Have anxiety or depression	336 (50.5)

*11 chronic diseases included physician diagnosed liver disease, lung disease, peptic ulcer disease, renal disease, arthritis, chronic back pain, diabetes, neurological disorder including stroke, cancer, allergy, and depression.

Table 2 shows that 14.0% of the respondents reported using Chinese herbal therapy and 71.3% reported using western drug to control blood pressure at the time of the interview. The rate of Chinese herbal therapy use varied by sex (13.6% for males than 14.2% for females), education (8.2% for illiteracy, 13.8% for elementary, and 20.5% for junior high or higher), and quality of life (for example, 4.6% for having problems with washing and dressing problem and 15.0% for no such problem).

**Table 2:** Rate of Chinese herbal therapy and western drug use* (%) among patients with diagnosed hypertension.

	Herb users (%)	Western drug users (%)	Non-users (%)	p-value
Overall	93(14.0)	474(71.3)	98(14.7)	
By sex				
Male	36(13.6)	173(65.6)	55(20.8)	0.001
Female	57(14.2)	301(75.1)	43(10.7)	
By age (years)				
30–49	15(15.6)	58(60.4)	23(24.0)	0.046
50–64	57(14.7)	281(72.6)	49(12.7)	
≥65	21(11.5)	135(74.2)	26(14.3)	
By employment				
Farmer	81 (14.1)	410 (71.4)	83(14.5)	0.870
Others	12 (13.2)	64 (70.3)	15(16.5)	
By education				
Illiteracy	13 (8.2)	133(83.6)	13(8.2)	0.000
Elementary	49 (13.8)	253(71.3)	53(14.9)	
Junior high or higher	31 (20.5)	88(58.3)	32(21.2)	
By marital status				
Married	84 (14.7)	407 (71.4)	79 (13.9)	0.157
Others	9 (9.5)	67 (70.5)	19 (20.0)	
By presence of any one of 11 chronic diseases**				
Yes	59 (15.5)	268 (70.3)	54 (14.7)	0.419
No	34 (12.0)	206 (72.5)	44 (15.5)	
By EuroQoL5				
Have walking problems	15 (9.1)	129 (78.2)	21 (12.7)	0.054
No walking problems	78 (15.6)	345 (69.0)	77 (15.4)	
Have washing and dressing problems	4 (4.6)	61 (84.7)	7 (9.7)	0.023
No washing and dressing problems	89 (15.0)	413 (69.6)	91 (15.4)	
Have usual activity problems	21 (13.7)	115 (75.2)	17 (11.1)	0.332
No usual activity problems	72 (14.1)	359 (70.1)	81 (15.8)	
Have pain or discomfort	49 (12.5)	294 (75.0)	49 (12.5)	0.037
No pain or discomfort	44 (16.1)	180 (65.9)	49 (18.0)	
Have anxiety or depression	41 (12.2)	247 (73.5)	48 (14.3)	0.348
No anxiety or depression	52 (15.8)	227 (69.0)	50 (15.2)	

* Use was defined based on response to the question: "How do you control your blood pressure now?" The multiple choices were western drug, Chinese herbal therapy, diet, acupuncture, physical activity, others (required to specify) and none. Chinese herbal therapy users and western drug users were mutually exclusive. Non-users were those who did not use Chinese herbal therapy or western drug.

**11 chronic diseases included physician diagnosed heart disease, liver disease, peptic ulcer disease, renal disease, arthritis, chronic back pain, diabetes, neurological disorder including stroke, cancer, allergy, and depression.

The mean of blood pressure level was similar between Chinese herbal therapy users and western drug users. Blood pressure was under control for 22.6% of the Chinese herbal therapy users and 22.4% of the western drug users (Table 3). Beliefs about Chinese herbal therapy were not significantly different among three groups of Chinese herbal therapy users, western drug users and non-users. More Chinese herbal therapy users than western drug users knew that high blood pressure could cause strokes (48.4% vs. 35.7%). The level of adherence to medicine was similar between Chinese herbal therapy users than western drug users (80.6% vs. 81.2% at low level. Table 4). Only 28.0% of the Chinese herbal therapy users and 30.8% of the western drug users did not stop taking medication sometimes when they felt that blood pressure was under control. The low adherence level is related to low level of hypertension knowledge (Pearson’s correlation coefficient = 0.184, P<0.05).

**Table 3:** Blood pressure under control, beliefs and hypertension knowledge among Chinese herbal therapy and western drug users* with diagnosed hypertension.

	Herb users N (% of 93)	Western drug users N (% of 474)	Non-users N (% of 98)	p value
**Blood pressure measurements**				
Systolic blood pressure measurements (mmHg)(Mean ± SD)	146.87±21.39	147.16±20.2	151.1±19.8	0.200
Diastolic blood pressure measurements (mmHg)(Mean ± SD)	91.37±12.37	90.93±13.08	95.66±13.75	0.005
Blood pressure under control(systolic pressure <140 mmHg and diastolic pressure <90 mmHg)	21 (22.6)	106 (22.4)	10 (10.2)	0.022
**Agree or some agree on the following statement (beliefs)**				
Some Chinese herbal therapists have secret therapies that have been passed down from generation to generation.	50 (53.8)	231(48.7)	47 (48.0)	0.65
In general, Chinese herbal remedies have less bad side effects than prescription western drugs.	58 (63.4)	260 (54.9)	57 (58.2)	0.38
Chinese herbal remedies have been scientifically tested to be effective.	64 (68.8)	317 (66.9)	67 (68.4)	0.91
Chinese herbalists treat patients with a holistic approach.	56 (60.2)	285 (60.1)	57 (58.2)	0.93
Disease is a result of ‘Yin and Yang’ disharmony.	40 (43.0)	199 (42.0)	40 (40.8)	0.95
Trust Chinese herbalist	60 (64.5)	294 (62.0)	64 (65.3)	0.78
**Correct answers to the following hypertension knowledge**				
If someone’s blood pressure is 120/80, it is normal.	65 (69.9)	301 (63.5)	58 (59.2)	0.299
If someone’s blood pressure is 160/100, it is hypertension.	73 (78.5)	336 (70.9)	61 (62.2)	0.047
High blood pressure can cause strokes.	45 (48.4)	169 (35.7)	29 (29.6)	0.020
High blood pressure can cause heart attacks.	45 (48.4)	189 (39.9)	25 (25.5)	0.004
High blood pressure can cause kidney problems.	27 (22.5)	81 (17.1)	12 (12.2)	0.006
High blood pressure can cause cancer problems.	6 (6.45)	14 (2.95)	3(3.06)	0.234
Hypertension usually lasts for the rest of the life.	48 (51.6)	218 (46.0)	31 (31.6)	0.012
Losing weight usually makes blood pressure go down.	61 (65.6)	248 (52.3)	47 (48.0)	0.031
Eating less salt usually makes blood pressure go down.	70 (75.3)	348 (73.4)	60 (61.2)	0.037
People with high blood pressure should take their medicine everyday.	57 (61.3)	283 (59.7)	38 (38.8)	0.002

* Use was defined based on response to the question: "How do you control your blood pressure now?" The multiple choices were western drug, Chinese herbal therapy, diet, acupuncture, physical activity, others (required to specify) and none. Chinese herbal therapy users and western drug users were mutually exclusive. Non-users were those who did not use Chinese herbal therapy or western drug.

**Table 4:** Adherence rate of Chinese herbal therapy and western drug among patients with diagnosed hypertension.

Adherence Items (answer)	Herb users* 93(%)	Western drug users 474 (%)	P value
#1	27 (29.0)	125 (26.4)	0.59
#2	41 (44.1)	175 (37.0)	0.19
#3	53 (57.0)	255 (53.8)	0.57
#4	45 (48.4)	205 (43.5)	0.36
#5	57 (61.3)	302 (64.1)	0.66
#6	26 (28.0)	146(30.8)	0.59
#7	31(33.3)	168 (35.4)	0.70
#8	75 (80.7)	350 (73.8)	0.17
Adherence level**			
Low (score <6)	75 (80.6)	385 (81.2)	0.72
Med (score 6–<8)	18 (19.4)	86 (18.1)	
High (score = 8)	0	3(0.6)	

* Use was defined based on response to the question: "How do you control your blood pressure now?" The multiple choices were western drug, Chinese herbal therapy, diet, acupuncture, physical activity, others (required to specify) and none. Chinese herbal therapy users and western drug users were mutually exclusive.

**Permission was obtained for adopting ^*©*^MMAS-8 (Morisky Medication Adherence Scale, 8-Items).[21] 1 score for each correct answer.

After adjustment for independent variables, only education was associated with Chinese herbal therapy use (Table 5). Respondents who were higher education level (adjusted odds ratio 2.6 for junior high school or higher, for elementary school relative to illiteracy) or better quality of life (risk adjusted odds ratio: 0.44 for having a problem on EQ5 vs. no problem) were more likely to use Chinese herbal therapy.

**Table 5:** Adjusted odds ratio (OR) and 95% confidence interval (95% CI) for factors associated with Chinese herbal therapy use* among patients with diagnosed hypertension.

Variable	OR (95% CI)
Male vs female	0.73 (0.44–1.21)
Age (years)	
30–49	1
50–64	1.25 (0.64–2.45)
≥65	1.28 (0.58–2.83)
Married	1.28 (0.59–2.78)
Education	
Illiteracy	1.0
Elementary school	1.55 (0.79–3.04)
Junior high school or higher	2.64 (1.21–5.79)
Presence of any one of 11 chronic diseases**	1.30 (0.79–2.12)
Having a problem based on EuroQol-5	0.44 (0.26–0.74)
Hypertension knowledge	
If someone’s blood pressure is 120/80, it is normal.	0.96 (0.54–1.69)
If someone’s blood pressure is 160/100, it is hypertension.	1.20 (0.62–2.31)
High blood pressure can cause strokes.	1.61 (0.94–2.78)
High blood pressure can cause heart attacks.	0.97 (0.55–1.72)
High blood pressure can cause kidney problems.	1.58 (0.866–2.89)
High blood pressure can cause cancer problems.	1.52 (0.538–4.31)
Hypertension usually lasts for the rest of the life.	1.20 (0.72–1.99)
Losing weight usually makes blood pressure go down.	1.55 (0.90–2.67)
Eating less salt usually makes blood pressure go down.	0.73 (0.39–1.36)
People with high blood pressure should take their medicine everyday.	0.86 (0.50–1.48)
Treatment adherence: Middle or high (score 6–8) vs low (score <6)	1.07 (0.59–1.94)

* Use was defined based on response to the question: "How do you control your blood pressure now?" The multiple choices were western drug, Chinese herbal therapy, diet, acupuncture, physical activity, others (required to specify) and none. Chinese herbal therapy users and western drug users were mutually exclusive. Baseline is non-Chinese herbal users.

**11 chronic diseases included physician diagnosed heart disease, liver disease, peptic ulcer disease, renal disease, arthritis, chronic back pain, diabetes, neurological disorder including stroke, cancer, allergy, and depression.

## Discussion

The results of our face to face interview highlighted the following. First, only 14.0% of the patients with diagnosed hypertension controlled their blood pressure using Chinese herbal therapies and 71.3% used western drugs. Second, the factors associated with Chinese herbal therapy use were education level and quality of life. Third, beliefs about Chinese herbal therapy were not associated with modality of hypertension management. Fourth, the medicine adherence level was similar between Chinese herbal therapy users and western drug users. Many patients stopped taking medicine when their blood pressure was under control.

The rate of Chinese herbal therapy use to control blood pressure was low, only 14.0%, which could be explained by disease, personal and cultural factors [23]. One potential reason for the low rate is that blood pressure could be effectively controlled by western drugs. Another possible reason is that Chinese herbal therapy is not the first choice of treatment for patients. Conventional beliefs in Chinese are that Chinese herbal therapy is effective in treatment of chronic diseases but not effective in acute conditions. Patients generally visit western medical doctors first for diagnosis and treatment. When the western drugs prescribed or recommended by the doctors are not effective or cause side-effects, patients start to see Chinese herbal therapists [23]. Thus Chinese herbal therapy is commonly used as alterative or complementary therapies for chronic and incurable diseases by western drugs, such heart disease[24], cancer[25], inflammatory bowel disease[26], back problems and stroke[27–28].

The question remains: why do some patients with diagnosed hypertension still use Chinese herbal therapy? Many factors could play a role in the decision making, including previous experience with western drug, openness to Chinese herbal therapy, and cost associated with western drug and Chinese herbal therapy. In China, health insurance covers both western drug and Chinese herbal therapy. Cost of anti-hypertensive western drug and Chinese herbal therapy is similar. Thus, Chinese herbal therapy users may feel that anti-hypertensive western drug is ineffective, or they may be concerned about western drug side effects. In our sample, 63.4% of Chinese herbal therapy users and 54.9% western drug users agree that Chinese herbal therapies have less “bad side effects” than western drugs although the difference was not statistically significant. Chinese herbal therapy users are more likely to have higher education than non-users. Quan et al. [2] also reported that Chinese Canadians with high education levels were less likely to use herbal therapy compared with those with lower education. However, complementary therapy (including Chinese herbal therapy) users tend to be females, have a high-income or have high education level compared to non-users in general population of Western countries [29–31].

A high proportion of patients with hypertension stopped taking medication sometimes when they felt their blood pressure was under control (72.0% for Chinese herbal therapy users and 69.2% for western drug users). Astonishingly, only 61.3% of the Chinese herbal therapy users and 59.7% of the western drug users knew that they should take their medications everyday and 51.6% of the Chinese herbal therapy users and 46.0% of the western drug users understood that hypertension usually lasts for the rest of their life. Further, few patients had knowledge about the organ complications related to hypertension. These knowledge gaps are somewhat surprising as in rural areas primary care physicians provide hypertension education. A lack of understanding of the long term health outcomes of hypertension may lead to poor compliance with Chinese herbal therapy and western drug.

Surprisingly, our study did not find users of both Chinese herbal therapy and western drug at the same time for blood pressure control although TCM and western medicine is well integrated in China. The first possible explanation is that patients with hypertension might use western drug first. When they did not find effect[32], they might turn to herb as the last resort. The second possible explanation is that patients might concern interaction between Chinese herbal therapy and western drug and chose one therapy. The third possible explanation is that some patients might not report using both therapies.

This study has limitations. First, we conducted interviews in two counties in the Heilongjiang Province of China. These counties are relatively poor and our results could not be generalized to other regions without caution and studies. Second, we interviewed patients who came to the interview sites. If this convenience sample had better health knowledge and medication adherence than those who were not surveyed, our findings about hypertension knowledge and adherence level could be over-estimated. However, our study focused on comparison between Chinese herbal therapy and western drug users. Thus, the potential sampling bias is less likely to influence the risk adjusted odds ratios and our conclusions. Third, we missed people who were temporarily away from these villages for various reasons, such as working in urban areas. These people are relatively healthy and have relatively high education than those at the villages. Fourth, use of certain therapy is determined by multiple factors. We included a limited number of socio-demographic and clinical variables. However we did not collect detailed history of therapy use (such as duration of therapy use and specific type of therapy) because of potential recall bias. Some patients may discontinue and switch therapies. Fifth, our study could not inform reasons herbal therapy use and just characterize hypertensive patients who used herbal therapy due to cross-sectional study design.

## Conclusion

Only a small proportion of patients with hypertension in rural areas used Chinese herbal therapy to control their blood pressure. The use was associated with education level and quality of life. The majority of the patients used anti-hypertensive western drugs. Patients with hypertension in rural areas had low level of Chinese herbal therapy and western drug adherence and knowledge about hypertension complications. Education program should emphasize that patients with hypertension should not stop their medications when their blood pressure normalizes

## Supporting Information

S1 Table SAS statistics process for Tables 1–4. (PDF)

S2 Table SAS statistics process for Table 5. (PDF)
