# Correction: Chinese Herbal Therapy and Western Drug Use, Belief and Adherence for Hypertension Management in the Rural Areas of Heilongjiang Province, China

**DOI:** 10.1371/journal.pone.0187613

**Published:** 2017-11-01

**Authors:** The PLOS ONE Staff

## Notice of Republication

Table 4 was included in the article without full permission. The table was removed from the HTML and PDF versions of this article on July 6, 2017. Please download this article again to view the correct version. Readers can access the MMAS-8 scale through the original publication [2].
