# Supplementary material for: Chinese Herbal Therapy and Western Drug Use, Belief and Adherence for Hypertension Management in the Rural Areas of Heilongjiang Province, China
Source: PLoS One. 2015 Apr 29;10(4):e0123508. doi: 10.1371/journal.pone.0123508 (PMC4414607; doi:10.1371/journal.pone.0123508)
Supplement: S1 Table — (PDF) [file pone.0123508.s001.pdf]

Table 1

The FREQ Procedure

Sex	Frequency	Percent	Cumulative Frequency	Cumulative Percent
1	264	39.70	264	39.70
2	401	60.30	665	100.00

age_group	Frequency	Percent	Cumulative Frequency	Cumulative Percent
30-49	96	14.44	96	14.44
50-64	387	58.20	483	72.63
gt 65	182	27.37	665	100.00

marriage	Frequency	Percent	Cumulative Frequency	Cumulative Percent
0	95	14.29	95	14.29
1	570	85.71	665	100.00

Education	Frequency	Percent	Cumulative Frequency	Cumulative Percent
1	159	23.91	159	23.91
2	355	53.38	514	77.29
3	151	22.71	665	100.00

disease_11	Frequency	Percent	Cumulative Frequency	Cumulative Percent
0	284	42.71	284	42.71
1	381	57.29	665	100.00

Q1_Walking	Frequency	Percent	Cumulative Frequency	Cumulative Percent
0	500	75.19	500	75.19
1	165	24.81	665	100.00

Q2_Dressing	Frequency	Percent	Cumulative Frequency	Cumulative Percent
0	593	89.17	593	89.17
1	72	10.83	665	100.00

Q3_daily	Frequency	Percent	Cumulative Frequency	Cumulative Percent
0	512	76.99	512	76.99
1	153	23.01	665	100.00

Table 1

The FREQ Procedure

Q4_Pain	Frequency	Percent	Cumulative Frequency	Cumulative Percent
0	273	41.05	273	41.05
1	392	58.95	665	100.00

Q5_anxiety	Frequency	Percent	Cumulative Frequency	Cumulative Percent
0	329	49.47	329	49.47
1	336	50.53	665	100.00

Table 2

The FREQ Procedure

Drug_use	Frequency	Percent	Cumulative Frequency	Cumulative Percent
1	98	14.74	98	14.74
2	474	71.28	572	86.02
3	93	13.98	665	100.00

Frequency<br>Percent<br>Row Pct<br>Col Pct

Table of Drug_use by Sex			
	Sex		
Drug_use	1	2	Total
1	55<br>8.27<br>56.12<br>20.83	43<br>6.47<br>43.88<br>10.72	98<br>14.74
2	173<br>26.02<br>36.50<br>65.53	301<br>45.26<br>63.50<br>75.06	474<br>71.28
3	36<br>5.41<br>38.71<br>13.64	57<br>8.57<br>61.29<br>14.21	93<br>13.98
Total	264<br>39.70	401<br>60.30	665<br>100.00

Statistics for Table of Drug_use by Sex

Statistic	DF	Value	Prob
Chi-Square	2	13.1090	0.0014
Likelihood Ratio Chi-Square	2	12.8272	0.0016
Mantel-Haenszel Chi-Square	1	6.3235	0.0119
Phi Coefficient		0.1404	
Contingency Coefficient		0.1390	
Cramer's V		0.1404	

Sample Size = 665

Table 2

The FREQ Procedure

Frequency
Percent
Row Pct
Col Pct

Table of Drug_use by age_group				
	age_group			
Drug_use	30-49	50-64	gt 65	Total
1	23<br>3.46<br>23.47<br>23.96	49<br>7.37<br>50.00<br>12.66	26<br>3.91<br>26.53<br>14.29	98<br>14.74
2	58<br>8.72<br>12.24<br>60.42	281<br>42.26<br>59.28<br>72.61	135<br>20.30<br>28.48<br>74.18	474<br>71.28
3	15<br>2.26<br>16.13<br>15.63	57<br>8.57<br>61.29<br>14.73	21<br>3.16<br>22.58<br>11.54	93<br>13.98
Total	96<br>14.44	387<br>58.20	182<br>27.37	665<br>100.00

Statistics for Table of Drug_use by age_group

Statistic	DF	Value	Prob
Chi-Square	4	9.7119	0.0456
Likelihood Ratio Chi-Square	4	9.0034	0.0610
Mantel-Haenszel Chi-Square	1	0.1733	0.6772
Phi Coefficient		0.1208	
Contingency Coefficient		0.1200	
Cramer's V		0.0855	

Sample Size = 665

Frequency
Percent
Row Pct
Col Pct

Table of Drug_use by work			
	work		
Drug_use	Others	farmer	Total
1	15<br>2.26<br>15.31<br>16.48	83<br>12.48<br>84.69<br>14.46	98<br>14.74
2	64<br>9.62<br>13.50<br>70.33	410<br>61.65<br>86.50<br>71.43	474<br>71.28
3	12<br>1.80<br>12.90<br>13.19	81<br>12.18<br>87.10<br>14.11	93<br>13.98
Total	91<br>13.68	574<br>86.32	665<br>100.00

Table 2

The FREQ Procedure

Statistics for Table of Drug_use by work

Statistic	DF	Value	Prob
Chi-Square	2	0.2796	0.8695
Likelihood Ratio Chi-Square	2	0.2736	0.8721
Mantel-Haenszel Chi-Square	1	0.2374	0.6261
Phi Coefficient		0.0205	
Contingency Coefficient		0.0205	
Cramer's V		0.0205	

Sample Size = 665

Frequency<br>Percent<br>Row Pct<br>Col Pct

Table of Drug_use by Education				
	Education			
Drug_use	1	2	3	Total
1	13<br>1.95<br>13.27<br>8.18	53<br>7.97<br>54.08<br>14.93	32<br>4.81<br>32.65<br>21.19	98<br>14.74
2	133<br>20.00<br>28.06<br>83.65	253<br>38.05<br>53.38<br>71.27	88<br>13.23<br>18.57<br>58.28	474<br>71.28
3	13<br>1.95<br>13.98<br>8.18	49<br>7.37<br>52.69<br>13.80	31<br>4.66<br>33.33<br>20.53	93<br>13.98
Total	159<br>23.91	355<br>53.38	151<br>22.71	665<br>100.00

Statistics for Table of Drug_use by Education

Statistic	DF	Value	Prob
Chi-Square	4	24.3858	<.0001
Likelihood Ratio Chi-Square	4	24.9216	<.0001
Mantel-Haenszel Chi-Square	1	0.0126	0.9106
Phi Coefficient		0.1915	
Contingency Coefficient		0.1881	
Cramer's V		0.1354	

Sample Size = 665

Table 2

The FREQ Procedure

Frequency<br>Percent<br>Row Pct<br>Col Pct

Table of Drug_use by marriage			
	marriage		
Drug_use	0	1	Total
1	19<br>2.86<br>19.39<br>20.00	79<br>11.88<br>80.61<br>13.86	98<br>14.74
2	67<br>10.08<br>14.14<br>70.53	407<br>61.20<br>85.86<br>71.40	474<br>71.28
3	9<br>1.35<br>9.68<br>9.47	84<br>12.63<br>90.32<br>14.74	93<br>13.98
Total	95<br>14.29	570<br>85.71	665<br>100.00

Statistics for Table of Drug_use by marriage

Statistic	DF	Value	Prob
Chi-Square	2	3.7050	0.1568
Likelihood Ratio Chi-Square	2	3.7045	0.1569
Mantel-Haenszel Chi-Square	1	3.6819	0.0550
Phi Coefficient		0.0746	
Contingency Coefficient		0.0744	
Cramer's V		0.0746	

Sample Size = 665

Frequency<br>Percent<br>Row Pct<br>Col Pct

Table of Drug_use by disease_11			
	disease_11		
Drug_use	0	1	Total
1	44<br>6.62<br>44.90<br>15.49	54<br>8.12<br>55.10<br>14.17	98<br>14.74
2	206<br>30.98<br>43.46<br>72.54	268<br>40.30<br>56.54<br>70.34	474<br>71.28
3	34<br>5.11<br>36.56<br>11.97	59<br>8.87<br>63.44<br>15.49	93<br>13.98
Total	284<br>42.71	381<br>57.29	665<br>100.00

The FREQ Procedure

Statistics for Table of Drug_use by disease_11

Statistic	DF	Value	Prob
Chi-Square	2	1.7387	0.4192
Likelihood Ratio Chi-Square	2	1.7595	0.4149
Mantel-Haenszel Chi-Square	1	1.3218	0.2503
Phi Coefficient		0.0511	
Contingency Coefficient		0.0511	
Cramer's V		0.0511	

Sample Size = 665

Frequency<br>Percent<br>Row Pct<br>Col Pct

Table of Drug_use by Q1_Walking			
	Q1_Walking		
Drug_use	0	1	Total
1	77<br>11.58<br>78.57<br>15.40	21<br>3.16<br>21.43<br>12.73	98<br>14.74
2	345<br>51.88<br>72.78<br>69.00	129<br>19.40<br>27.22<br>78.18	474<br>71.28
3	78<br>11.73<br>83.87<br>15.60	15<br>2.26<br>16.13<br>9.09	93<br>13.98
Total	500<br>75.19	165<br>24.81	665<br>100.00

Statistics for Table of Drug_use by Q1_Walking

Statistic	DF	Value	Prob
Chi-Square	2	5.8272	0.0543
Likelihood Ratio Chi-Square	2	6.1863	0.0454
Mantel-Haenszel Chi-Square	1	0.6349	0.4256
Phi Coefficient		0.0936	
Contingency Coefficient		0.0932	
Cramer's V		0.0936	

Sample Size = 665

The FREQ Procedure

Frequency<br>Percent<br>Row Pct<br>Col Pct

Table of Drug_use by Q2_Dressing

Drug_use	Q2_Dressing 0	Q2_Dressing 1	Total
1	91<br>13.68<br>92.86<br>15.35	7<br>1.05<br>7.14<br>9.72	98<br>14.74
2	413<br>62.11<br>87.13<br>69.65	61<br>9.17<br>12.87<br>84.72	474<br>71.28
3	89<br>13.38<br>95.70<br>15.01	4<br>0.60<br>4.30<br>5.56	93<br>13.98
Total	593<br>89.17	72<br>10.83	665<br>100.00

Statistics for Table of Drug_use by Q2_Dressing

Statistic	DF	Value	Prob
Chi-Square	2	7.5275	0.0232
Likelihood Ratio Chi-Square	2	8.6757	0.0131
Mantel-Haenszel Chi-Square	1	0.3274	0.5672
Phi Coefficient		0.1064	
Contingency Coefficient		0.1058	
Cramer's V		0.1064	

Sample Size = 665

Frequency<br>Percent<br>Row Pct<br>Col Pct

Table of Drug_use by Q3_daily

Drug_use	Q3_daily 0	Q3_daily 1	Total
1	81<br>12.18<br>82.65<br>15.82	17<br>2.56<br>17.35<br>11.11	98<br>14.74
2	359<br>53.98<br>75.74<br>70.12	115<br>17.29<br>24.26<br>75.16	474<br>71.28
3	72<br>10.83<br>77.42<br>14.06	21<br>3.16<br>22.58<br>13.73	93<br>13.98
Total	512<br>76.99	153<br>23.01	665<br>100.00

The FREQ Procedure

Statistics for Table of Drug_use by Q3_daily

Statistic	DF	Value	Prob
Chi-Square	2	2.2031	0.3324
Likelihood Ratio Chi-Square	2	2.3164	0.3140
Mantel-Haenszel Chi-Square	1	0.7830	0.3762
Phi Coefficient		0.0576	
Contingency Coefficient		0.0575	
Cramer's V		0.0576	

Sample Size = 665

Frequency<br>Percent<br>Row Pct<br>Col Pct

Table of Drug_use by Q4_Pain			
	Q4_Pain		
Drug_use	0	1	Total
1	49<br>7.37<br>50.00<br>17.95	49<br>7.37<br>50.00<br>12.50	98<br>14.74
2	180<br>27.07<br>37.97<br>65.93	294<br>44.21<br>62.03<br>75.00	474<br>71.28
3	44<br>6.62<br>47.31<br>16.12	49<br>7.37<br>52.69<br>12.50	93<br>13.98
Total	273<br>41.05	392<br>58.95	665<br>100.00

Statistics for Table of Drug_use by Q4_Pain

Statistic	DF	Value	Prob
Chi-Square	2	6.6033	0.0368
Likelihood Ratio Chi-Square	2	6.5474	0.0379
Mantel-Haenszel Chi-Square	1	0.1877	0.6648
Phi Coefficient		0.0996	
Contingency Coefficient		0.0992	
Cramer's V		0.0996	

Sample Size = 665

The FREQ Procedure

Frequency<br>Percent<br>Row Pct<br>Col Pct

Table of Drug_use by Q5_anxiety			
	Q5_anxiety		
Drug_use	0	1	Total
1	50<br>7.52<br>51.02<br>15.20	48<br>7.22<br>48.98<br>14.29	98<br>14.74
2	227<br>34.14<br>47.89<br>69.00	247<br>37.14<br>52.11<br>73.51	474<br>71.28
3	52<br>7.82<br>55.91<br>15.81	41<br>6.17<br>44.09<br>12.20	93<br>13.98
Total	329<br>49.47	336<br>50.53	665<br>100.00

Statistics for Table of Drug_use by Q5_anxiety

Statistic	DF	Value	Prob
Chi-Square	2	2.1123	0.3478
Likelihood Ratio Chi-Square	2	2.1154	0.3473
Mantel-Haenszel Chi-Square	1	0.4186	0.5176
Phi Coefficient		0.0564	
Contingency Coefficient		0.0563	
Cramer's V		0.0564	

Sample Size = 665

Table 3

The MEANS Procedure

Drug_use	N Obs	Variable	N	Mean	Std Dev	Minimum	Maximum
1	98	Systolic<br>Diastolic	98<br>98	151.1020408<br>95.6632653	19.8078344<br>13.7482474	110.0000000<br>70.0000000	200.0000000<br>145.0000000
2	474	Systolic<br>Diastolic	474<br>474	147.1677215<br>90.9303797	20.2000260<br>13.0778853	100.0000000<br>60.0000000	220.0000000<br>135.0000000
3	93	Systolic<br>Diastolic	93<br>93	146.8763441<br>91.3763441	21.3935250<br>12.3747323	107.5000000<br>58.5000000	217.5000000<br>117.5000000

Table 3

The ANOVA Procedure

Class Level Information		
**Class**	**Levels**	**Values**
**Drug_use**	3	1 2 3

**Number of Observations Read**	665
**Number of Observations Used**	665

Table 3

The ANOVA Procedure

Dependent Variable: Systolic

Source	DF	Sum of Squares	Mean Square	F Value	Pr > F
**Model**	2	1331.5959	665.7979	1.61	0.2000
**Error**	662	273168.2237	412.6408		
**Corrected Total**	664	274499.8195			

R-Square	Coeff Var	Root MSE	Systolic Mean
0.004851	13.75263	20.31356	147.7068

Source	DF	Anova SS	Mean Square	F Value	Pr > F
**Drug_use**	2	1331.595861	665.797930	1.61	0.2000

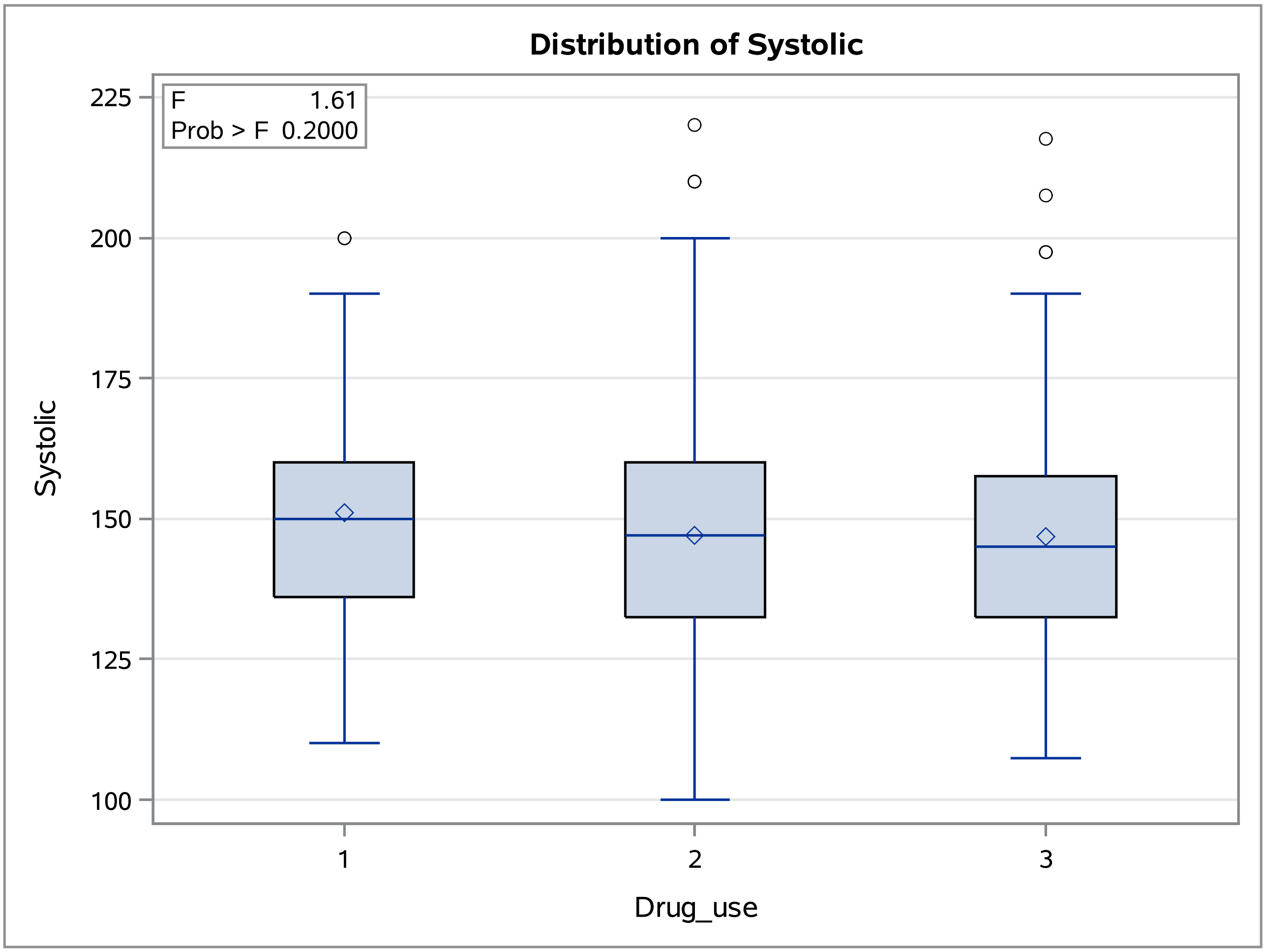

Table 3

The ANOVA Procedure

Class Level Information		
**Class**	**Levels**	**Values**
**Drug_use**	3	1 2 3

**Number of Observations Read**	665
**Number of Observations Used**	665

Table 3

The ANOVA Procedure

Dependent Variable: Diastolic

Source	DF	Sum of Squares	Mean Square	F Value	Pr > F
Model	2	1829.7682	914.8841	5.34	0.0050
Error	662	113320.4182	171.1789		
Corrected Total	664	115150.1865			

R-Square	Coeff Var	Root MSE	Diastolic Mean
0.015890	14.26928	13.08353	91.69023

Source	DF	Anova SS	Mean Square	F Value	Pr > F
Drug_use	2	1829.768222	914.884111	5.34	0.0050

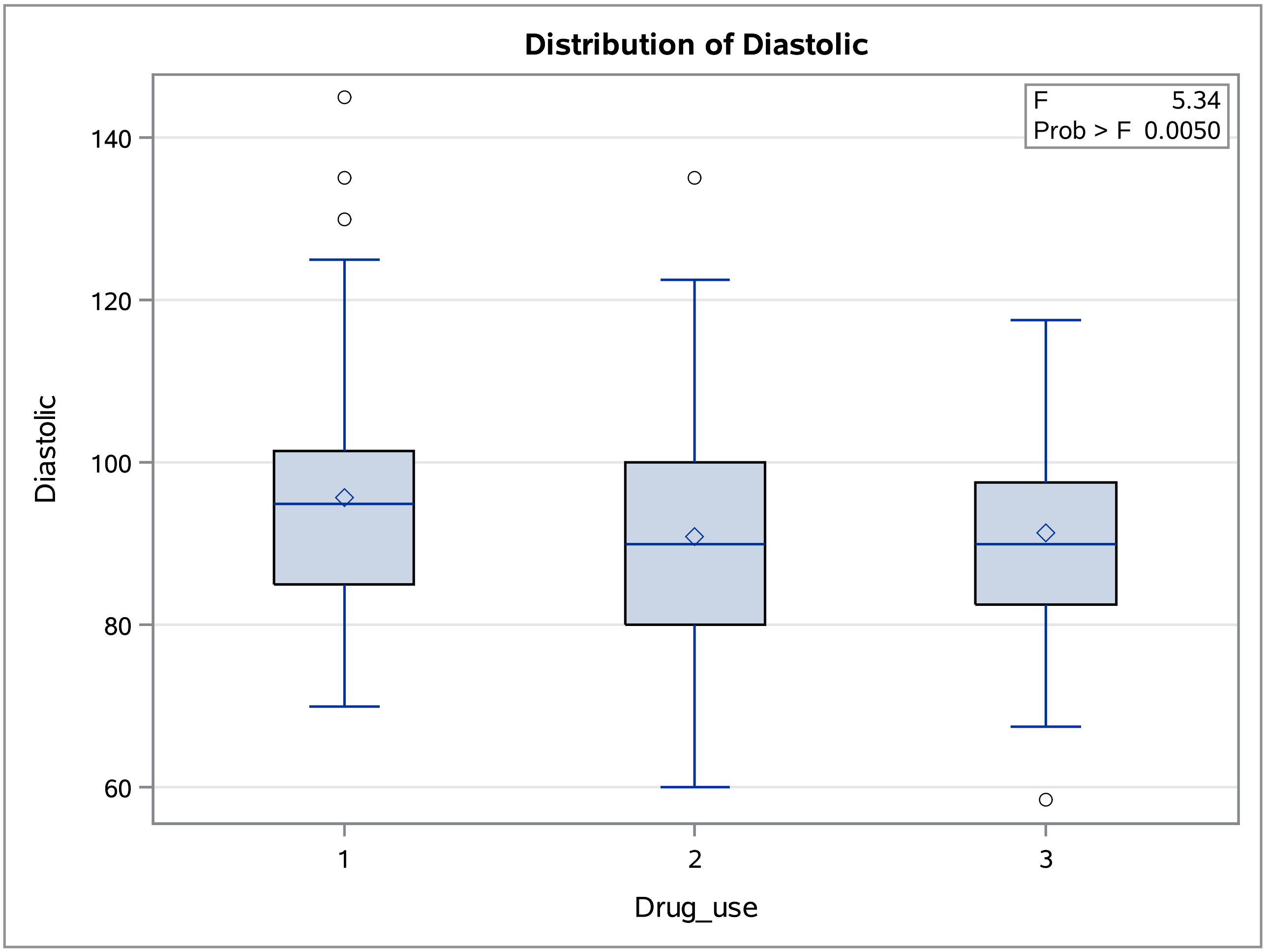

The FREQ Procedure

Frequency<br>Percent<br>Row Pct<br>Col Pct

Table of Drug_use by BP_undercontrol

Drug_use	BP_undercontrol 0	BP_undercontrol 1	Total
1	88<br>13.23<br>89.80<br>16.67	10<br>1.50<br>10.20<br>7.30	98<br>14.74
2	368<br>55.34<br>77.64<br>69.70	106<br>15.94<br>22.36<br>77.37	474<br>71.28
3	72<br>10.83<br>77.42<br>13.64	21<br>3.16<br>22.58<br>15.33	93<br>13.98
Total	528<br>79.40	137<br>20.60	665<br>100.00

Statistics for Table of Drug_use by BP_undercontrol

Statistic	DF	Value	Prob
Chi-Square	2	7.5986	0.0224
Likelihood Ratio Chi-Square	2	8.7056	0.0129
Mantel-Haenszel Chi-Square	1	4.6262	0.0315
Phi Coefficient		0.1069	
Contingency Coefficient		0.1063	
Cramer's V		0.1069	

Sample Size = 665

Frequency<br>Percent<br>Row Pct<br>Col Pct

Table of Drug_use by Belief_generation

Drug_use	Belief_generation 0	Belief_generation 1	Total
1	51<br>7.67<br>52.04<br>15.13	47<br>7.07<br>47.96<br>14.33	98<br>14.74
2	243<br>36.54<br>51.27<br>72.11	231<br>34.74<br>48.73<br>70.43	474<br>71.28
3	43<br>6.47<br>46.24<br>12.76	50<br>7.52<br>53.76<br>15.24	93<br>13.98
Total	337<br>50.68	328<br>49.32	665<br>100.00

Table 3

The FREQ Procedure

Statistics for Table of Drug_use by Belief_generation

Statistic	DF	Value	Prob
Chi-Square	2	0.8723	0.6465
Likelihood Ratio Chi-Square	2	0.8727	0.6464
Mantel-Haenszel Chi-Square	1	0.6250	0.4292
Phi Coefficient		0.0362	
Contingency Coefficient		0.0362	
Cramer's V		0.0362	

Sample Size = 665

Frequency<br>Percent<br>Row Pct<br>Col Pct

Table of Drug_use by Belief_sideeffect			
	Belief_sideeffect		
Drug_use	0	1	Total
1	41<br>6.17<br>41.84<br>14.14	57<br>8.57<br>58.16<br>15.20	98<br>14.74
2	214<br>32.18<br>45.15<br>73.79	260<br>39.10<br>54.85<br>69.33	474<br>71.28
3	35<br>5.26<br>37.63<br>12.07	58<br>8.72<br>62.37<br>15.47	93<br>13.98
Total	290<br>43.61	375<br>56.39	665<br>100.00

Statistics for Table of Drug_use by Belief_sideeffect

Statistic	DF	Value	Prob
Chi-Square	2	1.9314	0.3807
Likelihood Ratio Chi-Square	2	1.9483	0.3775
Mantel-Haenszel Chi-Square	1	0.3102	0.5776
Phi Coefficient		0.0539	
Contingency Coefficient		0.0538	
Cramer's V		0.0539	

Sample Size = 665

The FREQ Procedure

Frequency
Percent
Row Pct
Col Pct

Table of Drug_use by Belief_scientific			
	Belief_scientific		
Drug_use	0	1	Total
1	31<br>4.66<br>31.63<br>14.29	67<br>10.08<br>68.37<br>14.96	98<br>14.74
2	157<br>23.61<br>33.12<br>72.35	317<br>47.67<br>66.88<br>70.76	474<br>71.28
3	29<br>4.36<br>31.18<br>13.36	64<br>9.62<br>68.82<br>14.29	93<br>13.98
Total	217<br>32.63	448<br>67.37	665<br>100.00

Statistics for Table of Drug_use by Belief_scientific

Statistic	DF	Value	Prob
Chi-Square	2	0.1852	0.9116
Likelihood Ratio Chi-Square	2	0.1860	0.9112
Mantel-Haenszel Chi-Square	1	0.0032	0.9547
Phi Coefficient		0.0167	
Contingency Coefficient		0.0167	
Cramer's V		0.0167	

Sample Size = 665

Frequency
Percent
Row Pct
Col Pct

Table of Drug_use by Belief_holistic			
	Belief_holistic		
Drug_use	0	1	Total
1	41<br>6.17<br>41.84<br>15.36	57<br>8.57<br>58.16<br>14.32	98<br>14.74
2	189<br>28.42<br>39.87<br>70.79	285<br>42.86<br>60.13<br>71.61	474<br>71.28
3	37<br>5.56<br>39.78<br>13.86	56<br>8.42<br>60.22<br>14.07	93<br>13.98
Total	267<br>40.15	398<br>59.85	665<br>100.00

The FREQ Procedure

Statistics for Table of Drug_use by Belief_holistic

Statistic	DF	Value	Prob
Chi-Square	2	0.1363	0.9341
Likelihood Ratio Chi-Square	2	0.1358	0.9344
Mantel-Haenszel Chi-Square	1	0.0864	0.7688
Phi Coefficient		0.0143	
Contingency Coefficient		0.0143	
Cramer's V		0.0143	

Sample Size = 665

Frequency<br>Percent<br>Row Pct<br>Col Pct

Table of Drug_use by Belief_YinYang			
	Belief_YinYang		
Drug_use	0	1	Total
1	58<br>8.72<br>59.18<br>15.03	40<br>6.02<br>40.82<br>14.34	98<br>14.74
2	275<br>41.35<br>58.02<br>71.24	199<br>29.92<br>41.98<br>71.33	474<br>71.28
3	53<br>7.97<br>56.99<br>13.73	40<br>6.02<br>43.01<br>14.34	93<br>13.98
Total	386<br>58.05	279<br>41.95	665<br>100.00

Statistics for Table of Drug_use by Belief_YinYang

Statistic	DF	Value	Prob
Chi-Square	2	0.0949	0.9537
Likelihood Ratio Chi-Square	2	0.0949	0.9536
Mantel-Haenszel Chi-Square	1	0.0945	0.7586
Phi Coefficient		0.0119	
Contingency Coefficient		0.0119	
Cramer's V		0.0119	

Sample Size = 665

Table 3

The FREQ Procedure

Frequency<br>Percent<br>Row Pct<br>Col Pct

Table of Drug_use by Belief_Trust

Drug_use	Belief_Trust 0	Belief_Trust 1	Total
1	34<br>5.11<br>34.69<br>13.77	64<br>9.62<br>65.31<br>15.31	98<br>14.74
2	180<br>27.07<br>37.97<br>72.87	294<br>44.21<br>62.03<br>70.33	474<br>71.28
3	33<br>4.96<br>35.48<br>13.36	60<br>9.02<br>64.52<br>14.35	93<br>13.98
Total	247<br>37.14	418<br>62.86	665<br>100.00

Statistics for Table of Drug_use by Belief_Trust

Statistic	DF	Value	Prob
Chi-Square	2	0.5019	0.7781
Likelihood Ratio Chi-Square	2	0.5045	0.7770
Mantel-Haenszel Chi-Square	1	0.0165	0.8979
Phi Coefficient		0.0275	
Contingency Coefficient		0.0275	
Cramer's V		0.0275	

Sample Size = 665

Frequency<br>Percent<br>Row Pct<br>Col Pct

Table of Drug_use by Q1_120_80

Drug_use	Q1_120_80 0	Q1_120_80 1	Total
1	40<br>6.02<br>40.82<br>16.60	58<br>8.72<br>59.18<br>13.68	98<br>14.74
2	173<br>26.02<br>36.50<br>71.78	301<br>45.26<br>63.50<br>70.99	474<br>71.28
3	28<br>4.21<br>30.11<br>11.62	65<br>9.77<br>69.89<br>15.33	93<br>13.98
Total	241<br>36.24	424<br>63.76	665<br>100.00

The FREQ Procedure

Statistics for Table of Drug_use by Q1_120_80

Statistic	DF	Value	Prob
Chi-Square	2	2.4155	0.2989
Likelihood Ratio Chi-Square	2	2.4438	0.2947
Mantel-Haenszel Chi-Square	1	2.3487	0.1254
Phi Coefficient		0.0603	
Contingency Coefficient		0.0602	
Cramer's V		0.0603	

Sample Size = 665

Frequency<br>Percent<br>Row Pct<br>Col Pct

Table of Drug_use by Q2_160_100			
	Q2_160_100		
Drug_use	0	1	Total
1	37<br>5.56<br>37.76<br>18.97	61<br>9.17<br>62.24<br>12.98	98<br>14.74
2	138<br>20.75<br>29.11<br>70.77	336<br>50.53<br>70.89<br>71.49	474<br>71.28
3	20<br>3.01<br>21.51<br>10.26	73<br>10.98<br>78.49<br>15.53	93<br>13.98
Total	195<br>29.32	470<br>70.68	665<br>100.00

Statistics for Table of Drug_use by Q2_160_100

Statistic	DF	Value	Prob
Chi-Square	2	6.1146	0.0470
Likelihood Ratio Chi-Square	2	6.1282	0.0467
Mantel-Haenszel Chi-Square	1	6.0879	0.0136
Phi Coefficient		0.0959	
Contingency Coefficient		0.0955	
Cramer's V		0.0959	

Sample Size = 665

Table 3

The FREQ Procedure

Frequency<br>Percent<br>Row Pct<br>Col Pct

Table of Drug_use by Q10_strokes			
	Q10_strokes		
Drug_use	0	1	Total
1	69<br>10.38<br>70.41<br>16.35	29<br>4.36<br>29.59<br>11.93	98<br>14.74
2	305<br>45.86<br>64.35<br>72.27	169<br>25.41<br>35.65<br>69.55	474<br>71.28
3	48<br>7.22<br>51.61<br>11.37	45<br>6.77<br>48.39<br>18.52	93<br>13.98
Total	422<br>63.46	243<br>36.54	665<br>100.00

Statistics for Table of Drug_use by Q10_strokes

Statistic	DF	Value	Prob
Chi-Square	2	7.8297	0.0199
Likelihood Ratio Chi-Square	2	7.7035	0.0212
Mantel-Haenszel Chi-Square	1	7.1661	0.0074
Phi Coefficient		0.1085	
Contingency Coefficient		0.1079	
Cramer's V		0.1085	

Sample Size = 665

Frequency<br>Percent<br>Row Pct<br>Col Pct

Table of Drug_use by Q7_heart_disease			
	Q7_heart_disease		
Drug_use	0	1	Total
1	73<br>10.98<br>74.49<br>17.98	25<br>3.76<br>25.51<br>9.65	98<br>14.74
2	285<br>42.86<br>60.13<br>70.20	189<br>28.42<br>39.87<br>72.97	474<br>71.28
3	48<br>7.22<br>51.61<br>11.82	45<br>6.77<br>48.39<br>17.37	93<br>13.98
Total	406<br>61.05	259<br>38.95	665<br>100.00

Table 3

The FREQ Procedure

Statistics for Table of Drug_use by Q7_heart_disease

Statistic	DF	Value	Prob
Chi-Square	2	11.0976	0.0039
Likelihood Ratio Chi-Square	2	11.4644	0.0032
Mantel-Haenszel Chi-Square	1	10.5921	0.0011
Phi Coefficient		0.1292	
Contingency Coefficient		0.1281	
Cramer's V		0.1292	

Sample Size = 665

Frequency<br>Percent<br>Row Pct<br>Col Pct

Table of Drug_use by Q9_renal			
	Q9_renal		
Drug_use	0	1	Total
1	86<br>12.93<br>87.76<br>15.78	12<br>1.80<br>12.24<br>10.00	98<br>14.74
2	393<br>59.10<br>82.91<br>72.11	81<br>12.18<br>17.09<br>67.50	474<br>71.28
3	66<br>9.92<br>70.97<br>12.11	27<br>4.06<br>29.03<br>22.50	93<br>13.98
Total	545<br>81.95	120<br>18.05	665<br>100.00

Statistics for Table of Drug_use by Q9_renal

Statistic	DF	Value	Prob
Chi-Square	2	10.1139	0.0064
Likelihood Ratio Chi-Square	2	9.4308	0.0090
Mantel-Haenszel Chi-Square	1	8.9409	0.0028
Phi Coefficient		0.1233	
Contingency Coefficient		0.1224	
Cramer's V		0.1233	

Sample Size = 665

Table 3

The FREQ Procedure

Frequency<br>Percent<br>Row Pct<br>Col Pct

Table of Drug_use by Q8_cancer

Drug_use	Q8_cancer 0	Q8_cancer 1	Total
1	95<br>14.29<br>96.94<br>14.80	3<br>0.45<br>3.06<br>13.04	98<br>14.74
2	460<br>69.17<br>97.05<br>71.65	14<br>2.11<br>2.95<br>60.87	474<br>71.28
3	87<br>13.08<br>93.55<br>13.55	6<br>0.90<br>6.45<br>26.09	93<br>13.98
Total	642<br>96.54	23<br>3.46	665<br>100.00

Statistics for Table of Drug_use by Q8_cancer

Statistic	DF	Value	Prob
Chi-Square	2	2.9034	0.2342
Likelihood Ratio Chi-Square	2	2.4304	0.2967
Mantel-Haenszel Chi-Square	1	1.5765	0.2093
Phi Coefficient		0.0661	
Contingency Coefficient		0.0659	
Cramer's V		0.0661	
WARNING: 33% of the cells have expected counts less than 5. Chi-Square may not be a valid test.			

Sample Size = 665

Frequency<br>Percent<br>Row Pct<br>Col Pct

Table of Drug_use by Q3_last

Drug_use	Q3_last 0	Q3_last 1	Total
1	67<br>10.08<br>68.37<br>18.21	31<br>4.66<br>31.63<br>10.44	98<br>14.74
2	256<br>38.50<br>54.01<br>69.57	218<br>32.78<br>45.99<br>73.40	474<br>71.28
3	45<br>6.77<br>48.39<br>12.23	48<br>7.22<br>51.61<br>16.16	93<br>13.98
Total	368<br>55.34	297<br>44.66	665<br>100.00

The FREQ Procedure

Statistics for Table of Drug_use by Q3_last

Statistic	DF	Value	Prob
Chi-Square	2	8.8885	0.0117
Likelihood Ratio Chi-Square	2	9.0908	0.0106
Mantel-Haenszel Chi-Square	1	7.8259	0.0052
Phi Coefficient		0.1156	
Contingency Coefficient		0.1148	
Cramer's V		0.1156	

Sample Size = 665

Frequency<br>Percent<br>Row Pct<br>Col Pct

Table of Drug_use by Q4_take_medicine			
	Q4_take_medicine		
Drug_use	0	1	Total
1	60<br>9.02<br>61.22<br>20.91	38<br>5.71<br>38.78<br>10.05	98<br>14.74
2	191<br>28.72<br>40.30<br>66.55	283<br>42.56<br>59.70<br>74.87	474<br>71.28
3	36<br>5.41<br>38.71<br>12.54	57<br>8.57<br>61.29<br>15.08	93<br>13.98
Total	287<br>43.16	378<br>56.84	665<br>100.00

Statistics for Table of Drug_use by Q4_take_medicine

Statistic	DF	Value	Prob
Chi-Square	2	15.3725	0.0005
Likelihood Ratio Chi-Square	2	15.2428	0.0005
Mantel-Haenszel Chi-Square	1	10.1685	0.0014
Phi Coefficient		0.1520	
Contingency Coefficient		0.1503	
Cramer's V		0.1520	

Sample Size = 665

Table 3

The FREQ Procedure

Frequency<br>Percent<br>Row Pct<br>Col Pct

Table of Drug_use by Q5_lostweight			
	Q5_lostweight		
Drug_use	0	1	Total
1	51<br>7.67<br>52.04<br>16.50	47<br>7.07<br>47.96<br>13.20	98<br>14.74
2	226<br>33.98<br>47.68<br>73.14	248<br>37.29<br>52.32<br>69.66	474<br>71.28
3	32<br>4.81<br>34.41<br>10.36	61<br>9.17<br>65.59<br>17.13	93<br>13.98
Total	309<br>46.47	356<br>53.53	665<br>100.00

Statistics for Table of Drug_use by Q5_lostweight

Statistic	DF	Value	Prob
Chi-Square	2	6.9402	0.0311
Likelihood Ratio Chi-Square	2	7.0558	0.0294
Mantel-Haenszel Chi-Square	1	5.8459	0.0156
Phi Coefficient		0.1022	
Contingency Coefficient		0.1016	
Cramer's V		0.1022	

Sample Size = 665

Frequency<br>Percent<br>Row Pct<br>Col Pct

Table of Drug_use by Q6_salt			
	Q6_salt		
Drug_use	0	1	Total
1	38<br>5.71<br>38.78<br>20.32	60<br>9.02<br>61.22<br>12.55	98<br>14.74
2	126<br>18.95<br>26.58<br>67.38	348<br>52.33<br>73.42<br>72.80	474<br>71.28
3	23<br>3.46<br>24.73<br>12.30	70<br>10.53<br>75.27<br>14.64	93<br>13.98
Total	187<br>28.12	478<br>71.88	665<br>100.00

Table 3

The FREQ Procedure

Statistics for Table of Drug_use by Q6_salt

Statistic	DF	Value	Prob
Chi-Square	2	6.5878	0.0371
Likelihood Ratio Chi-Square	2	6.2685	0.0435
Mantel-Haenszel Chi-Square	1	4.7804	0.0288
Phi Coefficient		0.0995	
Contingency Coefficient		0.0990	
Cramer's V		0.0995	

Sample Size = 665

The FREQ Procedure

Frequency<br>Percent<br>Row Pct<br>Col Pct

Table of Drug_use by A1_sometimesforget			
	A1_sometimesforget		
Drug_use	0	1	Total
2	349<br>61.55<br>73.63<br>84.10	125<br>22.05<br>26.37<br>82.24	474<br>83.60
3	66<br>11.64<br>70.97<br>15.90	27<br>4.76<br>29.03<br>17.76	93<br>16.40
Total	415<br>73.19	152<br>26.81	567<br>100.00

Statistics for Table of Drug_use by A1_sometimesforget

Statistic	DF	Value	Prob
Chi-Square	1	0.2806	0.5963
Likelihood Ratio Chi-Square	1	0.2768	0.5988
Continuity Adj. Chi-Square	1	0.1613	0.6879
Mantel-Haenszel Chi-Square	1	0.2801	0.5967
Phi Coefficient		0.0222	
Contingency Coefficient		0.0222	
Cramer's V		0.0222	

Fisher's Exact Test	
Cell (1,1) Frequency (F)	349
Left-sided Pr <= F	0.7470
Right-sided Pr >= F	0.3400
Table Probability (P)	0.0869
Two-sided Pr <= P	0.6096

Sample Size = 567

Table 3

The FREQ Procedure

Frequency<br>Percent<br>Row Pct<br>Col Pct

Table of Drug_use by A2_twoweeks			
	A2_twoweeks		
Drug_use	0	1	Total
2	299<br>52.73<br>63.08<br>85.19	175<br>30.86<br>36.92<br>81.02	474<br>83.60
3	52<br>9.17<br>55.91<br>14.81	41<br>7.23<br>44.09<br>18.98	93<br>16.40
Total	351<br>61.90	216<br>38.10	567<br>100.00

Statistics for Table of Drug_use by A2_twoweeks

Statistic	DF	Value	Prob
Chi-Square	1	1.6930	0.1932
Likelihood Ratio Chi-Square	1	1.6698	0.1963
Continuity Adj. Chi-Square	1	1.4028	0.2363
Mantel-Haenszel Chi-Square	1	1.6900	0.1936
Phi Coefficient		0.0546	
Contingency Coefficient		0.0546	
Cramer's V		0.0546	

Fisher's Exact Test	
Cell (1,1) Frequency (F)	299
Left-sided Pr <= F	0.9211
Right-sided Pr >= F	0.1185
Table Probability (P)	0.0397
Two-sided Pr <= P	0.2006

Sample Size = 567

Table 3

The FREQ Procedure

Frequency<br>Percent<br>Row Pct<br>Col Pct

Table of Drug_use by A3_feelworse			
	A3_feelworse		
Drug_use	0	1	Total
2	219<br>38.62<br>46.20<br>84.56	255<br>44.97<br>53.80<br>82.79	474<br>83.60
3	40<br>7.05<br>43.01<br>15.44	53<br>9.35<br>56.99<br>17.21	93<br>16.40
Total	259<br>45.68	308<br>54.32	567<br>100.00

Statistics for Table of Drug_use by A3_feelworse

Statistic	DF	Value	Prob
Chi-Square	1	0.3192	0.5721
Likelihood Ratio Chi-Square	1	0.3201	0.5715
Continuity Adj. Chi-Square	1	0.2035	0.6519
Mantel-Haenszel Chi-Square	1	0.3186	0.5724
Phi Coefficient		0.0237	
Contingency Coefficient		0.0237	
Cramer's V		0.0237	

Fisher's Exact Test	
Cell (1,1) Frequency (F)	219
Left-sided Pr <= F	0.7509
Right-sided Pr >= F	0.3267
Table Probability (P)	0.0776
Two-sided Pr <= P	0.6490

Sample Size = 567

The FREQ Procedure

Frequency<br>Percent<br>Row Pct<br>Col Pct

Table of Drug_use by A4_travel			
	A4_travel		
Drug_use	0	1	Total
2	269<br>47.44<br>56.75<br>84.86	205<br>36.16<br>43.25<br>82.00	474<br>83.60
3	48<br>8.47<br>51.61<br>15.14	45<br>7.94<br>48.39<br>18.00	93<br>16.40
Total	317<br>55.91	250<br>44.09	567<br>100.00

Statistics for Table of Drug_use by A4_travel

Statistic	DF	Value	Prob
Chi-Square	1	0.8326	0.3615
Likelihood Ratio Chi-Square	1	0.8290	0.3626
Continuity Adj. Chi-Square	1	0.6373	0.4247
Mantel-Haenszel Chi-Square	1	0.8312	0.3619
Phi Coefficient		0.0383	
Contingency Coefficient		0.0383	
Cramer's V		0.0383	

Fisher's Exact Test	
Cell (1,1) Frequency (F)	269
Left-sided Pr <= F	0.8477
Right-sided Pr >= F	0.2121
Table Probability (P)	0.0598
Two-sided Pr <= P	0.3637

Sample Size = 567

Table 3

The FREQ Procedure

Frequency<br>Percent<br>Row Pct<br>Col Pct

Table of Drug_use by A5_yersterday			
	A5_yersterday		
Drug_use	0	1	Total
2	172<br>30.34<br>36.29<br>82.69	302<br>53.26<br>63.71<br>84.12	474<br>83.60
3	36<br>6.35<br>38.71<br>17.31	57<br>10.05<br>61.29<br>15.88	93<br>16.40
Total	208<br>36.68	359<br>63.32	567<br>100.00

Statistics for Table of Drug_use by A5_yersterday

Statistic	DF	Value	Prob
Chi-Square	1	0.1965	0.6576
Likelihood Ratio Chi-Square	1	0.1953	0.6585
Continuity Adj. Chi-Square	1	0.1060	0.7447
Mantel-Haenszel Chi-Square	1	0.1961	0.6579
Phi Coefficient		-0.0186	
Contingency Coefficient		0.0186	
Cramer's V		-0.0186	

Fisher's Exact Test	
Cell (1,1) Frequency (F)	172
Left-sided Pr <= F	0.3701
Right-sided Pr >= F	0.7141
Table Probability (P)	0.0842
Two-sided Pr <= P	0.7242

Sample Size = 567

Table 3

The FREQ Procedure

Frequency<br>Percent<br>Row Pct<br>Col Pct

Table of Drug_use by A6_Stop			
	A6_Stop		
Drug_use	0	1	Total
2	328<br>57.85<br>69.20<br>83.04	146<br>25.75<br>30.80<br>84.88	474<br>83.60
3	67<br>11.82<br>72.04<br>16.96	26<br>4.59<br>27.96<br>15.12	93<br>16.40
Total	395<br>69.66	172<br>30.34	567<br>100.00

Statistics for Table of Drug_use by A6_Stop

Statistic	DF	Value	Prob
Chi-Square	1	0.2977	0.5853
Likelihood Ratio Chi-Square	1	0.3014	0.5830
Continuity Adj. Chi-Square	1	0.1783	0.6728
Mantel-Haenszel Chi-Square	1	0.2972	0.5857
Phi Coefficient		-0.0229	
Contingency Coefficient		0.0229	
Cramer's V		-0.0229	

Fisher's Exact Test	
Cell (1,1) Frequency (F)	328
Left-sided Pr <= F	0.3398
Right-sided Pr >= F	0.7462
Table Probability (P)	0.0860
Two-sided Pr <= P	0.6236

Sample Size = 567

The FREQ Procedure

Frequency<br>Percent<br>Row Pct<br>Col Pct

Table of Drug_use by A7_hassled

Drug_use	A7_hassled 0	A7_hassled 1	Total
2	306<br>53.97<br>64.56<br>83.15	168<br>29.63<br>35.44<br>84.42	474<br>83.60
3	62<br>10.93<br>66.67<br>16.85	31<br>5.47<br>33.33<br>15.58	93<br>16.40
Total	368<br>64.90	199<br>35.10	567<br>100.00

Statistics for Table of Drug_use by A7_hassled

Statistic	DF	Value	Prob
Chi-Square	1	0.1519	0.6967
Likelihood Ratio Chi-Square	1	0.1529	0.6958
Continuity Adj. Chi-Square	1	0.0734	0.7864
Mantel-Haenszel Chi-Square	1	0.1516	0.6970
Phi Coefficient		-0.0164	
Contingency Coefficient		0.0164	
Cramer's V		-0.0164	

Fisher's Exact Test	
Cell (1,1) Frequency (F)	306
Left-sided Pr <= F	0.3961
Right-sided Pr >= F	0.6923
Table Probability (P)	0.0884
Two-sided Pr <= P	0.7232

Sample Size = 567

Table 3

The FREQ Procedure

Frequency<br>Percent<br>Row Pct<br>Col Pct

Table of Drug_use by A8_oftenforget			
	A8_oftenforget		
Drug_use	0	1	Total
2	124<br>21.87<br>26.16<br>87.32	350<br>61.73<br>73.84<br>82.35	474<br>83.60
3	18<br>3.17<br>19.35<br>12.68	75<br>13.23<br>80.65<br>17.65	93<br>16.40
Total	142<br>25.04	425<br>74.96	567<br>100.00

Statistics for Table of Drug_use by A8_oftenforget

Statistic	DF	Value	Prob
Chi-Square	1	1.9182	0.1661
Likelihood Ratio Chi-Square	1	2.0084	0.1564
Continuity Adj. Chi-Square	1	1.5728	0.2098
Mantel-Haenszel Chi-Square	1	1.9148	0.1664
Phi Coefficient		0.0582	
Contingency Coefficient		0.0581	
Cramer's V		0.0582	

Fisher's Exact Test	
Cell (1,1) Frequency (F)	124
Left-sided Pr <= F	0.9380
Right-sided Pr >= F	0.1031
Table Probability (P)	0.0412
Two-sided Pr <= P	0.1912

Sample Size = 567

Table 3

The FREQ Procedure

Frequency<br>Percent<br>Row Pct<br>Col Pct

Table of Drug_use by Adherence				
	Adherence			
Drug_use	Hig	Low	Med	Total
**2**	3<br>0.53<br>0.63<br>100.00	385<br>67.90<br>81.22<br>83.70	86<br>15.17<br>18.14<br>82.69	474<br>83.60
**3**	0<br>0.00<br>0.00<br>0.00	75<br>13.23<br>80.65<br>16.30	18<br>3.17<br>19.35<br>17.31	93<br>16.40
**Total**	3<br>0.53	460<br>81.13	104<br>18.34	567<br>100.00

Statistics for Table of Drug_use by Adherence

Statistic	DF	Value	Prob
Chi-Square	2	0.6540	0.7211
Likelihood Ratio Chi-Square	2	1.1394	0.5657
Mantel-Haenszel Chi-Square	1	0.1682	0.6818
Phi Coefficient		0.0340	
Contingency Coefficient		0.0339	
Cramer's V		0.0340	
WARNING: 33% of the cells have expected counts less than 5. Chi-Square may not be a valid test.			

Sample Size = 567

The LOGISTIC Procedure

Model Information	
Data Set	WORK.MYDATA
Response Variable	Herbal_vs_others
Number of Response Levels	2
Model	binary logit
Optimization Technique	Fisher's scoring

Number of Observations Read	665
Number of Observations Used	665

Response Profile		
Ordered Value	Herbal_vs_others	Total Frequency
1	0	572
2	1	93

Probability modeled is Herbal_vs_others=0.

Class Level Information			
Class	Value	Design Variables	
Sex	1	1	
	2	0	
Education	1	0	0
	2	1	0
	3	0	1
age_group	30-49	0	0
	50-64	1	0
	gt 65	0	1

Model Convergence Status
Convergence criterion (GCONV=1E-8) satisfied.

Model Fit Statistics		
Criterion	Intercept Only	Intercept and Covariates
AIC	540.238	549.125
SC	544.738	643.620
-2 Log L	538.238	507.125

Table 3

The LOGISTIC Procedure

Testing Global Null Hypothesis: BETA=0			
Test	Chi-Square	DF	Pr > ChiSq
Likelihood Ratio	31.1135	20	0.0537
Score	31.4628	20	0.0494
Wald	29.0819	20	0.0862

Type 3 Analysis of Effects			
Effect	DF	Wald Chi-Square	Pr > ChiSq
age_group	2	0.4234	0.8092
Sex	1	0.3844	0.5353
Education	2	6.9333	0.0312
heart_disease	1	3.4072	0.0649
Renal	1	0.5777	0.4472
diabetes	1	0.2086	0.6479
Cancer	1	0.0974	0.7549
Stroke_neurological_	1	0.1964	0.6577
Q1_120_80	1	0.0017	0.9675
Q2_160_100	1	0.2378	0.6258
Q10_strokes	1	1.6518	0.1987
Q7_heart_disease	1	0.1746	0.6760
Q9_renal	1	3.1826	0.0744
Q8_cancer	1	0.4426	0.5059
Q3_last	1	0.3673	0.5445
Q4_take_medicine	1	0.2031	0.6522
Q5_lostweight	1	3.2679	0.0706
Q6_salt	1	0.9181	0.3380

Analysis of Maximum Likelihood Estimates						
Parameter		DF	Estimate	Standard Error	Wald Chi-Square	Pr > ChiSq
Intercept		1	3.0831	0.5304	33.7814	<.0001
age_group	50-64	1	-0.2178	0.3456	0.3973	0.5285
age_group	gt 65	1	-0.1344	0.4031	0.1111	0.7389
Sex	1	1	0.1611	0.2598	0.3844	0.5353
Education	2	1	-0.5304	0.3441	2.3757	0.1232
Education	3	1	-1.0339	0.3977	6.7584	0.0093
heart_disease		1	-0.4649	0.2518	3.4072	0.0649

The LOGISTIC Procedure

Analysis of Maximum Likelihood Estimates						
Parameter		DF	Estimate	Standard Error	Wald Chi-Square	Pr > ChiSq
Renal		1	-0.2843	0.3741	0.5777	0.4472
diabetes		1	-0.1445	0.3165	0.2086	0.6479
Cancer		1	0.1615	0.5174	0.0974	0.7549
Stroke_neurological_		1	0.1979	0.4466	0.1964	0.6577
Q1_120_80		1	0.0120	0.2950	0.0017	0.9675
Q2_160_100		1	-0.1640	0.3364	0.2378	0.6258
Q10_strokes		1	-0.3557	0.2768	1.6518	0.1987
Q7_heart_disease		1	0.1240	0.2967	0.1746	0.6760
Q9_renal		1	-0.5470	0.3066	3.1826	0.0744
Q8_cancer		1	-0.3533	0.5311	0.4426	0.5059
Q3_last		1	-0.1566	0.2583	0.3673	0.5445
Q4_take_medicine		1	0.1240	0.2751	0.2031	0.6522
Q5_lostweight		1	-0.4975	0.2752	3.2679	0.0706
Q6_salt		1	0.3045	0.3178	0.9181	0.3380

Odds Ratio Estimates				
Effect		Point Estimate	95% Wald Confidence Limits	
age_group	50-64 vs 30-49	0.804	0.409	1.583
age_group	gt 65 vs 30-49	0.874	0.397	1.926
Sex	1 vs 2	1.175	0.706	1.955
Education	2 vs 1	0.588	0.300	1.155
Education	3 vs 1	0.356	0.163	0.775
heart_disease		0.628	0.383	1.029
Renal		0.753	0.362	1.567
diabetes		0.865	0.465	1.609
Cancer		1.175	0.426	3.240
Stroke_neurological_		1.219	0.508	2.925
Q1_120_80		1.012	0.568	1.804
Q2_160_100		0.849	0.439	1.641
Q10_strokes		0.701	0.407	1.205
Q7_heart_disease		1.132	0.633	2.025
Q9_renal		0.579	0.317	1.055
Q8_cancer		0.702	0.248	1.989
Q3_last		0.855	0.515	1.419

Table 3

The LOGISTIC Procedure

Odds Ratio Estimates			
Effect	Point Estimate	95% Wald Confidence Limits	
Q4_take_medicine	1.132	0.660	1.941
Q5_lostweight	0.608	0.355	1.043
Q6_salt	1.356	0.727	2.528

Association of Predicted Probabilities and Observed Responses			
Percent Concordant	66.9	Somers' D	0.346
Percent Discordant	32.3	Gamma	0.348
Percent Tied	0.8	Tau-a	0.083
Pairs	53196	c	0.673
