# Supplementary material for: Chinese Herbal Therapy and Western Drug Use, Belief and Adherence for Hypertension Management in the Rural Areas of Heilongjiang Province, China
Source: PLoS One. 2015 Apr 29;10(4):e0123508. doi: 10.1371/journal.pone.0123508 (PMC4414607; doi:10.1371/journal.pone.0123508)
Supplement: S2 Table — (PDF) [file pone.0123508.s002.pdf]

The LOGISTIC Procedure

Model Information	
**Data Set**	WORK.MYDATA
**Response Variable**	Herbal_vs_others
**Number of Response Levels**	2
**Model**	binary logit
**Optimization Technique**	Fisher's scoring

**Number of Observations Read**	665
**Number of Observations Used**	665

Response Profile		
**Ordered Value**	**Herbal_vs_others**	**Total Frequency**
**1**	0	572
**2**	1	93

Probability modeled is Herbal_vs_others=1.

Class Level Information			
**Class**	**Value**	**Design Variables**	
**Sex**	**1**	1	
	**2**	0	
**Education**	**1**	0	0
	**2**	1	0
	**3**	0	1
**age_group**	**30-49**	0	0
	**50-64**	1	0
	**gt 65**	0	1
**Adherence_new**	**Low**	0	
	**Med**	1	

Model Convergence Status
Convergence criterion (GCONV=1E-8) satisfied.

The LOGISTIC Procedure

Model Fit Statistics		
Criterion	Intercept Only	Intercept and Covariates
AIC	540.238	542.008
SC	544.738	632.003
-2 Log L	538.238	502.008

Testing Global Null Hypothesis: BETA=0			
Test	Chi-Square	DF	Pr > ChiSq
Likelihood Ratio	36.2306	19	0.0099
Score	36.3374	19	0.0096
Wald	33.1819	19	0.0229

Type 3 Analysis of Effects			
Effect	DF	Wald Chi-Square	Pr > ChiSq
Sex	1	1.5024	0.2203
age_group	2	0.4680	0.7914
marriage	1	0.3929	0.5308
Education	2	6.2432	0.0441
disease_11	1	1.0801	0.2987
EQ5problem	1	9.6900	0.0019
Q1_120_80	1	0.0230	0.8793
Q2_160_100	1	0.2830	0.5947
Q10_strokes	1	2.9896	0.0838
Q7_heart_disease	1	0.0102	0.9195
Q9_renal	1	2.2264	0.1357
Q8_cancer	1	0.6264	0.4287
Q3_last	1	0.4905	0.4837
Q4_take_medicine	1	0.2911	0.5895
Q5_lostweight	1	2.5457	0.1106
Q6_salt	1	1.0049	0.3161
Adherence_new	1	0.0471	0.8282

The LOGISTIC Procedure

Analysis of Maximum Likelihood Estimates						
**Parameter**		**DF**	**Estimate**	**Standard Error**	**Wald Chi-Square**	**Pr > ChiSq**
**Intercept**		1	-2.6152	0.6385	16.7763	<.0001
**Sex**	**1**	1	-0.3182	0.2596	1.5024	0.2203
**age_group**	**50-64**	1	0.2227	0.3445	0.4177	0.5181
**age_group**	**gt 65**	1	0.2475	0.4047	0.3741	0.5408
**marriage**		1	0.2449	0.3907	0.3929	0.5308
**Education**	**2**	1	0.4391	0.3433	1.6359	0.2009
**Education**	**3**	1	0.9708	0.4003	5.8800	0.0153
**disease_11**		1	0.2601	0.2503	1.0801	0.2987
**EQ5problem**		1	-0.8190	0.2631	9.6900	0.0019
**Q1_120_80**		1	-0.0441	0.2905	0.0230	0.8793
**Q2_160_100**		1	0.1787	0.3360	0.2830	0.5947
**Q10_strokes**		1	0.4786	0.2768	2.9896	0.0838
**Q7_heart_disease**		1	-0.0293	0.2902	0.0102	0.9195
**Q9_renal**		1	0.4582	0.3071	2.2264	0.1357
**Q8_cancer**		1	0.4202	0.5309	0.6264	0.4287
**Q3_last**		1	0.1816	0.2593	0.4905	0.4837
**Q4_take_medicine**		1	-0.1498	0.2777	0.2911	0.5895
**Q5_lostweight**		1	0.4400	0.2758	2.5457	0.1106
**Q6_salt**		1	-0.3185	0.3177	1.0049	0.3161
**Adherence_new**	**Med**	1	0.0658	0.3032	0.0471	0.8282

Odds Ratio Estimates			
**Effect**	**Point Estimate**	**95% Wald Confidence Limits**	
**Sex 1 vs 2**	0.727	0.437	1.210
**age_group 50-64 vs 30-49**	1.249	0.636	2.454
**age_group gt 65 vs 30-49**	1.281	0.579	2.832
**marriage**	1.277	0.594	2.748
**Education 2 vs 1**	1.551	0.792	3.040
**Education 3 vs 1**	2.640	1.205	5.786
**disease_11**	1.297	0.794	2.118
**EQ5problem**	0.441	0.263	0.738

The LOGISTIC Procedure

Odds Ratio Estimates			
Effect	Point Estimate	95% Wald Confidence Limits	
Q1_120_80	0.957	0.541	1.691
Q2_160_100	1.196	0.619	2.310
Q10_strokes	1.614	0.938	2.776
Q7_heart_disease	0.971	0.550	1.715
Q9_renal	1.581	0.866	2.887
Q8_cancer	1.522	0.538	4.310
Q3_last	1.199	0.721	1.993
Q4_take_medicine	0.861	0.500	1.483
Q5_lostweight	1.553	0.904	2.666
Q6_salt	0.727	0.390	1.356
Adherence_new Med vs Low	1.068	0.590	1.935

Association of Predicted Probabilities and Observed Responses			
Percent Concordant	67.8	Somers' D	0.363
Percent Discordant	31.5	Gamma	0.366
Percent Tied	0.7	Tau-a	0.088
Pairs	53196	c	0.682
